# BRAF mutation in multiple primary cancer with colorectal cancer and stomach cancer

**DOI:** 10.1093/gastro/got004

**Published:** 2013-04-05

**Authors:** Seung-Hyun Lee, Byung-Kwon Ahn, Sung-Uhn Baek, Hee-Kyung Chang

**Affiliations:** Department of Surgery and Pathology, Kosin University College of Medicine, Busan, Korea

**Keywords:** multiple primary cancer, colorectal cancer, stomach cancer, BRAF mutation

## Abstract

**Aims:** Recently, BRAF mutation testing has been introduced as a marker in differentiating Lynch syndrome from sporadic colorectal cancers or in predicting colorectal cancers with worse prognosis. Individuals with hereditary predisposition to cancer development are at an increased risk of developing multiple primary cancers. The purpose of this study is to identify mutation in the BRAF gene in multiple primary cancers with colorectal cancer and stomach cancer.

**Methods:** BRAF mutation was analysed in 45 patients with colorectal cancer and stomach cancer, synchronously or metachronously.

**Results:** Mean age was 64.07 years (range: 47–83 years). For the colorectal cancer, tumors were located at the sigmoid colon in eight patients (17.8%) and at the rectum in 22 patients (48.9%). Twenty-three patients (51.1%) had synchronous cancer. Four patients (8.9%) had family members with cancer. BRAF mutation was identified in three patients (6.7%). All three of these patients had metachronous cancers. The colorectal cancers were located in the sigmoid colon (1 patient) and the rectum (2 patients).

**Conclusions:** BRAF mutation rate was low in the multiple primary cancer with colorectal cancer and stomach cancer. With only BRAF gene study, it was not possible to identify any correlation with family history of colorectal cancer. Further study means considering other genes – MSI, MSH2, MLH1, MSH6.

## INTRODUCTION

Colorectal cancer is the one of the most common cancers worldwide. With the advancements in screening, diagnostic technique, treatment modality and prolonged survival, the incidence of multiple primary cancers with colorectal cancer has increasing pattern with long-term follow-up [1]. In multiple primary cancers with colorectal cancer, the stomach has been reported as the most common extracolonic organ in the Korean population [2–4].

Lynch syndrome is at an elevated risk of developing a second primary colorectal cancer as well as at an increased risk for extra-colonic malignancies including gastric, small bowel, urological tract, ovarian, pancreatic and brain cancers [5]. In multiple primary cancers with colorectal cancer, differentiating familiar colorectal cancer from sporadic colorectal cancer is important for family screening surveillance. Some genetic analyses, such as microsatellite instability (MSI), methylation of MLH1, MSH2, MSH6 and BRAF, have been proposed as a screening program for Lynch syndrome [6–8].

Recently, BRAF mutation testing has been introduced as a marker in differentiating sporadic colorectal cancers from Lynch syndrome [8]: colorectal cancers having a BRAF mutation are very unlikely to have Lynch syndrome, whereas those without a BRAF mutation should be further evaluated using other tests [9]. Also it has a role in predicting colorectal cancers with worse prognosis and in identifying colorectal cancers lacking BRAF mutation that are more likely to respond to epidermal growth factor receptor inhibitor therapy [10, 11].

Individuals with multiple primary cancers with colorectal cancer are at an increased risk of having hereditary predisposition to cancer development [1]. The stomach has been reported as the most common extracolonic organ in the Korean population [2–4]. According to other studies about multiple primary cancer with colorectal cancer in Korea, the most common extracolonic site (organs other than the colon) with multiple primary cancer was the stomach.

## MATERIALS AND METHODS

BRAF mutation was analysed in 45 patients with colorectal cancer and stomach cancer, synchronously or metachronously.

### DNA extraction

Formalin-fixed, paraffin-embedded tissues from colon cancer were studied. A hematoxylin and eosin-stained (H&E stained) section from each tissue sample was examined by an experienced pathologist to confirm histology. From each sample, one section was H&E stained as a reference and further sections (10 μm) were used for manual microdissection under a microscope to obtain at least 70–80% tumor cells

Before the extraction, tissue sections were deparaffinized using two washes with xylene and a final wash in absolute ethanol. The resulting samples were incubated in 300 µl TL buffer and 30 µl proteinase K at 55°C with shaking and left overnight to ensure that they were completely lysed. DNA extraction was performed with a High Pure PCR template preparation kit (Roche, Germany).

### Peptide nucleic acid-mediated clamping polymerase chain reaction for detection of BRAF mutation

The assays for the detection of BRAF V600E mutation in exon 15 were obtained through the PNAClamp™ BRAF mutation detection kit (PANAGENE, Inc. Daejeon, Korea). All reactions were done in 20 ul volumes using template DNA, primer and peptide nucleic acid (PNA) probe set, and SYBR® Green polymerase chain reaction (PCR) master mix. All reagents needed are included with the kit. Real-time PNA-mediated clamping PCR was performed using a CFX 96 (Real-Time PCR Detection System BIO-RAD CFX96 by Bio-Rad Laboratories, Inc., USA). PCR cycling conditions were a 5 min hold at 94°C followed by 40 cycles of 94°C for 30 sec, 70°C for 20 sec, 63°C for 30 sec and 72°C for 30 sec. Detection of mutation in the BRAF gene was possible using one-step PNA-mediated real-time PCR clamping.

**Figure 1:**
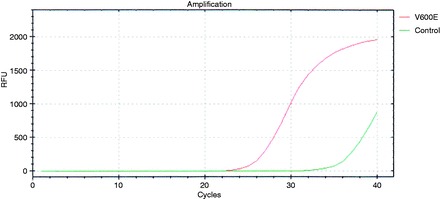
Peptide nucleic acid (PNA)-mediated clamping PCR analysis of BRAF level using SYBR® Green detection. We calculated the delta Ct (threshold cycle) (ΔCt) value as follows, ensuring that the sample and standard Ct values were from the tested sample and clamping control sample: [Standard Ct]-[Sample Ct] = ΔCt. The cut-off ΔCt was defined as 2 for the BRAF mutation. The ΔCt was 10.57. The SYBR® Green amplification plot shows the mutant type of BRAF mutation.

In this assay, PNA probes and DNA primers are used together in the clamping reaction. Positive signals are detected by intercalation of SYBR® Green fluorescent dye. The PNA probe sequence, which is complementary to wild-type DNA, suppresses amplification of wild-type targets, thereby enhancing preferential amplification of mutant sequences by competitively inhibiting DNA primer binding to wild-type DNA. PCR efficiency was determined by measuring the threshold cycle (Ct) value. We obtained Ct values for the control and mutation assays by observing the SYBR® Green amplification plots. We calculated the delta Ct (ΔCt) value as follows, ensuring that the Sample and Standard Ct values were from the tested sample and clamping control sample: [Standard Ct]-[Sample Ct] = ΔCt. The cut-off ΔCt was defined as 2 for the BRAF mutation (Figure 1).

## RESULTS

Mean age of the patients with multiple primary cancer, colorectal cancer and stomach cancer was 64.07 years (range: 47–83 years). Colorectal cancers were located at the cecum in 4 patients, the ascending colon in 5 patients, the transverse colon in 3 patients, the descending colon in 3 patients, the sigmoid colon in 8 patients and the rectum in 22 patients (48.9%). Twenty-three patients (51.1%) had synchronous cancer. Six patients (13.3%) had triple multiple primary cancers. Three patients had double cancers in the colorectum. The other three patients had cancers in the thyroid (papillary carcinoma), the lung (squamous cell carcinoma) and the kidney (renal cell carcinoma). Four patients (8.9%) had family members with cancer. Two patients had duodenal adenoma. BRAF mutation was identified in 3 patients (6.7%) (Table 1). The expression of BRAF mutation was not correlated with age, tumor location, diagnostic interval, tumor numbers or family cancer history. For positive BRAF mutation, the colorectal cancers were located in the sigmoid colon (1 patient) and the rectum (2 patients). All of the three patients had metachronous cancers (Table 2).

**Table 1:** Characteristics of multiple primary cancers with colorectal cancer and stomach cancer

Demographics	Cases (%)
Enrolled	45
Sex	
Male	32 (71.1)
Female	13 (28.9)
Age (years)*	64.07 (47–83)
<50	4 (8.9)
≥50	41 (91.1)
Tumor location of colorectal cancer	
Cecum	4 (8.9)
Ascending colon	5 (11.1)
Transverse colon	3 (6.7)
Descending colon	3 (6.7)
Sigmoid	8 (17.8)
Rectum	22 (48.9)
Stage of colorectal cancer	
0	1 (2.2)
1	6 (13.3)
2	15 (33.3)
3	18 (40.0)
4	5 (11.1)
Multiple primary cancer	
Synchronous	23 (51.1)
Metachronous	22 (48.9)
Number of multiple primary cancer	
Double	39 (86.7)
Triple	6 (13.3)
BARF mutation	
Negative	42 (93.3)
Positive	3 (6.7)

*Values are mean (range); other values in parentheses are percentages.

**Table 2:** Expression of BRAF mutation according to clinical factors of multiple primary cancers with colorectal cancer and stomach cancer

	BRAF mutation		*P* value
	Positive (%)	Negative (%)	
Age			0.751
<50	0	4 (100)	
≥50	3 (7.3)	38 (92.7)	
Location of cancers			0.557
Right side colon*	0	12 (100)	
Left side colon^†^	1 (9.1)	10 (90.9)	
Rectum	2 (9.1)	20 (90.9)	
Diagnostic interval			0.109
Synchronous	0	23 (100)	
Metachronous	3 (13.6)	19 (86.4)	
Number of cancers			0.644
Double	3 (7.7)	36 (92.3)	
Triple	0	6 (100)	

*Right side colon, including appendix, cecum, ascending colon, hepatic flexure and transverse colon; †Left side colon, including splenic flexure, descending colon and sigmoid colon.

## DISCUSSION

With advancements in screening, diagnostic technique, treatment modality and prolonged survival, the incidence of multiple primary cancers with colorectal cancer has increasing pattern with long-term follow-up [1]. The stomach has been reported as the most common extracolonic organ with multiple primary cancers in the Korean population [2–4]. In our previous study, the incidence of multiple primary cancers in patients with colorectal cancer was 3.0%. The most common extra-colonic site of the multiple primary cancers among colorectal cancer patients (1.6%) was the stomach [4].

Individuals with multiple primary cancers with colorectal cancer are at an increased risk of having hereditary predisposition to cancer development [1]. Lynch syndrome is at an elevated risk of developing a second primary colorectal cancer as well as at an increased risk for extra-colonic malignancies, including gastric, small bowel, urological tract, ovarian, pancreatic and brain cancers [5, 12]. In multiple primary cancers with colorectal cancer, differentiating familiar colorectal cancer from sporadic colorectal cancer is important for family screening surveillance. The most effective strategy for the diagnosis of Lynch syndrome is the recording of a family history using the Amsterdam criteria [13]. However, this family history information is often not available. Some genetic analyses, such as microsatellite instability (MSI), methylation of MLH1, MSH2, MSH6 and BRAF, have been proposed as a screening program for Lynch syndrome [6–8]. Some colorectal cancers with Lynch syndrome-like pedigrees remain mutation-negative in a DNA mismatch repair (MMR) gene analysis [14].

Recently, BRAF mutation testing has been introduced as a marker in differentiating sporadic colorectal cancers from Lynch syndrome [8]. Colorectal cancers having a BRAF mutation are very unlikely to have Lynch syndrome, whereas those without a BRAF mutation should be further evaluated using other tests [9]. Gudgeon *et al.* [9] reported the primary outcomes of simulation models in a cohort of 100 colorectal cancer cases for Lynch syndrome screening protocol. They suggested the screening model using immuno-histochemistry (IHC) of MMR protein with methylation the MLH1 promoter and then BRAF analysis as the standard protocol for Lynch syndrome screening. However, in their study, sensitivity of screening protocol with IHC of MMR protein and BRAF mutation was 92.79%, not lower than that of protocol using all three tests.

In the present study, multiple primary cancer patients with colorectal- and stomach cancer were selected for BRAF analysis. This is the first paper reporting BRAF mutation in a Korean population with colorectal- and stomach cancer. Three (6.7%) out of 45 cases showed positive. The BRAF mutation rate of this present study was low compare with that reported in western studies [15–16].

Shen *et al. *[17] reported 1.7% of BRAF mutation in a Chinese population with colorectal cancers. The mutation rate was also very low compare with that reported in western studies. They suggested two factors for the reason of low mutation rate. First, they only analysed mutations at codon 600 in exon 15 of the BRAF gene, not at other sites. Second, ethnic differences might exist between Chinese and western populations. We also analysed only BRAF V600E mutation in exon 15. The BRAF mutation rate in this present study was higher than that of Chinese population [17–18]. It might be the cause of the difference in the study population of colorectal cancer with or without multiple primary cancers.

In this present study, BRAF mutation was not correlated with sex, age (>50 years), location of colorectal cancers (right side, left side and rectum), stage of colorectal cancers or the number of multiple primary cancers (double; triple) (Table 2).

In conclusion, BRAF mutation rate was low in multiple primary cancer with colorectal cancer and stomach cancer in a Korean population. To use BRAF mutation as a marker for indentifying hereditary predisposition, more experiments of BRAF mutation or other genetic studies are necessary.

## FUNDING

This study was supported by a grant from Kosin University College of Medicine (2010).

**Conflict of interest:** none declared.
